# Molecular Docking Studies of Ortho-Substituted Phenols to Tyrosinase Helps Discern If a Molecule Can Be an Enzyme Substrate

**DOI:** 10.3390/ijms25136891

**Published:** 2024-06-23

**Authors:** María F. Montenegro, José A. Teruel, Pablo García-Molina, José Tudela, José Neptuno Rodríguez-López, Francisco García-Cánovas, Francisco García-Molina

**Affiliations:** 1 GENZ-Group of Research on Enzymology, Department of Biochemistry and Molecular Biology-A, Regional Campus of International Excellence “Campus Mare Nostrum”, University of Murcia, 30100 Murcia, Spain; fermontenegro@um.es (M.F.M.); pablo.garcia14@um.es (P.G.-M.); tudelaj@um.es (J.T.); neptuno@um.es (J.N.R.-L.); 2 Department of Biochemistry and Molecular Biology-A, Regional Campus of International Excellence “Campus Mare Nostrum”, University of Murcia, 30100 Murcia, Spain; teruel@um.es; 3 Department of Anatomía Patológica, Hospital General Universitario Reina Sofía, Av. Intendente Jorge Palacios, 1, 30003 Murcia, Spain; pacogm@um.es

**Keywords:** tyrosinase, substrates, inhibitors, ortho-substituted phenols

## Abstract

Phenolic compounds with a position ortho to the free phenolic hydroxyl group occupied can be tyrosinase substrates. However, ortho-substituted compounds are usually described as inhibitors. The mechanism of action of tyrosinase on monophenols is complex, and if they are ortho-substituted, it is more complicated. It can be shown that many of these molecules can become substrates of the enzyme in the presence of catalytic *o*-diphenol, MBTH, or in the presence of hydrogen peroxide. Docking studies can help discern whether a molecule can behave as a substrate or inhibitor of the enzyme. Specifically, phenols such as thymol, carvacrol, guaiacol, eugenol, isoeugenol, and ferulic acid are substrates of tyrosinase, and docking simulations to the active center of the enzyme predict this since the distance of the peroxide oxygen from the oxy-tyrosinase form to the ortho position of the phenolic hydroxyl is adequate for the electrophilic attack reaction that gives rise to hydroxylation occurring.

## 1. Introduction

Tyrosinase (EC 1.14.18.1) is a copper monooxygenase widely distributed throughout the phylogenetic scale: bacteria, fungi, plants, and animals. Basically, it catalyzes two types of reaction: the hydroxylation of monophenols to *o*-diphenols and the oxidation of *o*-diphenols to *o*-quinones, both using molecular oxygen as a co-substrate [1,2].

The products of the tyrosinase enzymatic reaction are *o*-quinones, which are unstable. In the case of tyrosinase from mammals, the physiological substrates are L-tyrosine and L-dopa. Through the action of tyrosinase on L-tyrosine, *o*-quinones are produced that chemically evolve following a fixed stoichiometry, which leads to the accumulation of *o*-diphenol (L-dopa) in the reaction medium; this causes the enzyme in its action on L-tyrosine to reach the steady state and the dopachrome product to accumulate after a period of delay according to a straight line. The delay period corresponds to the time that the action of tyrosinase takes to accumulate a certain concentration of *o-*diphenol in the medium [3].

**Figure 1:**
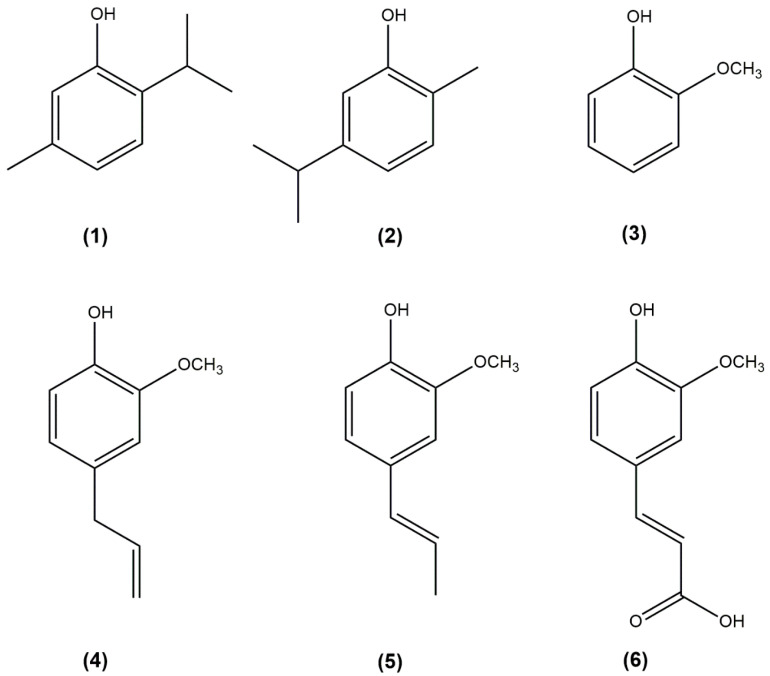
Structures of the compounds used in the present study. (**1**) thymol (2-isopropyl-5-methylphenol); (**2**) carvacrol (2-isopropyl-5-methylphenol); (**3**) guaiacol (2-metoxyphenol); (**4**) eugenol (4-allyl-2-metoxyphenol); (**5**) isoeugenol (2-metoxy-4-propenylphenol); and (**6**) ferulic acid (3-metoxy-4-hydroxycinnamic acid).

**Scheme 1:**
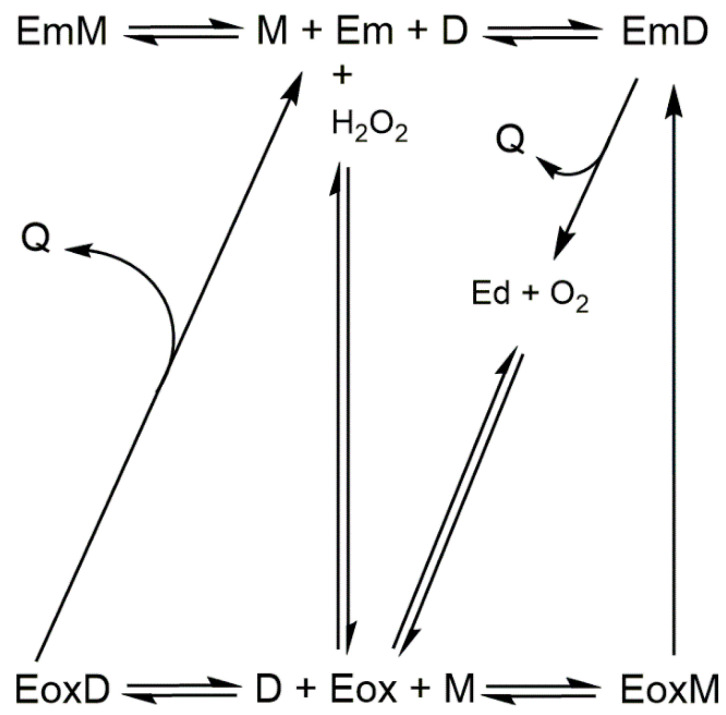
Action mechanism of tyrosinase on monophenol in the presence of hydrogen peroxide. Em, met-tyrosinase; Ed, deoxy-tyrosinase; Eox, oxy-tyrosinase; M, monophenol; D, *o*-diphenol; and Q, *o*-quinone.

In the case of ortho-substituted phenols (Figure 1), although the oxy form of tyrosinase is able to hydroxylate it in the ortho position of the phenolic hydroxyl and subsequently oxidize it to *o*-quinone, the evolution of this does not lead to the accumulation of *o*-diphenol in the medium. Apparently, the enzyme does not show activity on this compound; this case is similar to that of hydroquinone [4], oxyresveratrol [5], hexylresorcinol [6], p-coumaric [7], 4-n-butylresorcinol [8], and resorcinols [9]. The presence of hydrogen peroxide, which achieves the transformation of meta-tyrosinase into oxy-tyrosinase, shows that these compounds may be alternative substrates for tyrosinase (Scheme 1).

**Scheme 2:**
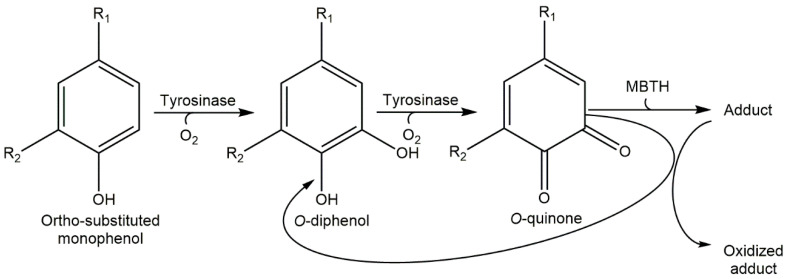
Enzymatic activity on ortho-substituted phenols in the presence of MBTH.

This transformation can also occur in the presence of an *o*-diphenol in catalytic quantities such as L-dopa; in this case, *met-*tyrosinase becomes *desoxy*-tyrosinase, and this can react with oxygen to give oxy-tyrosinase [1,2,10]. The formation of hydrogen peroxide in the melanin biosynthesis pathway has been demonstrated [11]. The presence of hydrogen peroxide could help to identify a molecule as an alternative substrate for tyrosinase [12]. On the other hand, the presence of a nucleophile such as MBTH can help with the accumulation of *o*-diphenol in the medium, which favors the transition from met-tyrosinase to oxy-tyrosinase (Scheme 2).

**Figure 2:**
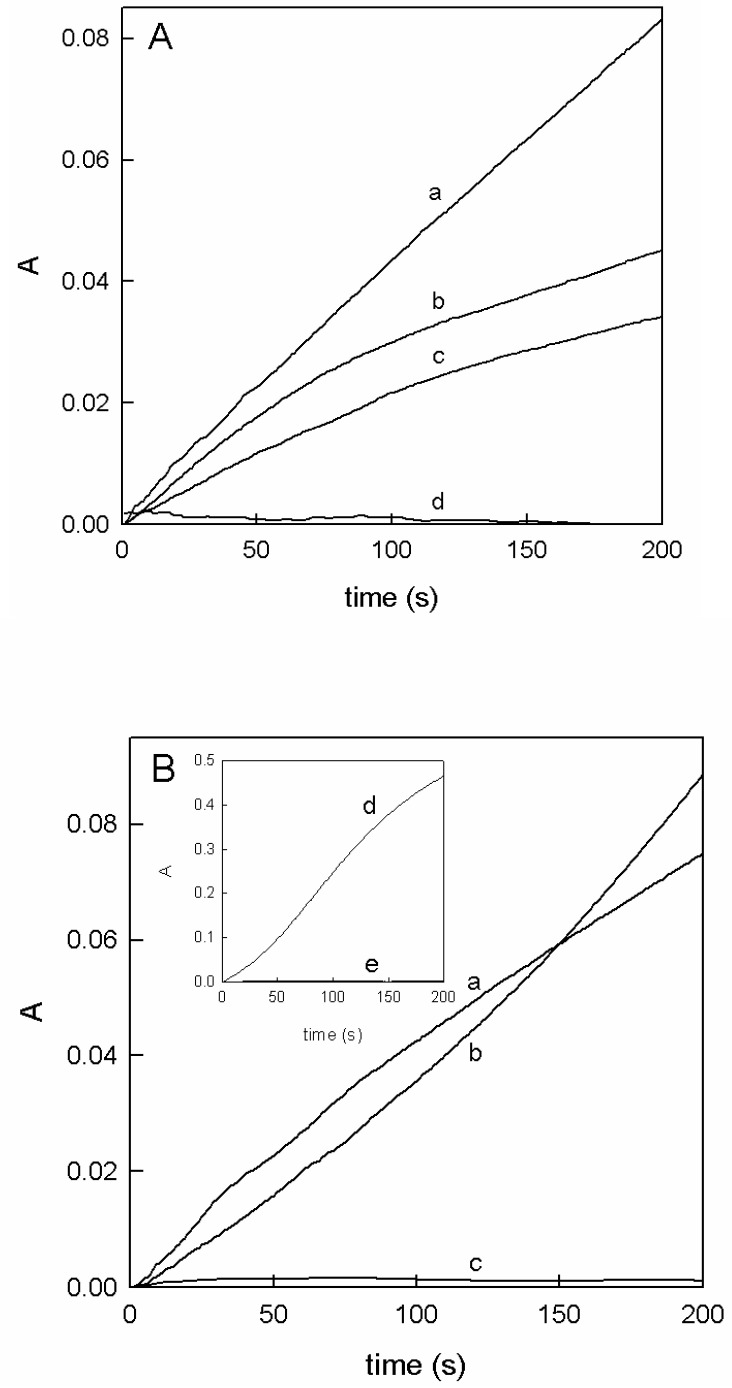
Action of tyrosinase on these monophenols. (**A**) Spectrophotometric recordings of the action of tyrosinase (0.1 mg/mL) on guaiacol following a 540 nm. Curve (a). Guaiacol 1.5 mM in the presence of MBTH 1 mM and N,N′ dimethylformamide 2% in 50 mM phosphate buffer (pH 7.0). Curve (b). Increase in absorbance followed at 437 nm due to the action of tyrosinase (0.1 mg/mL) on carvacrol 1.5 mM and 0.083 mM of (3I6MC) in 30 mM phosphate buffer (pH 7.0). Curve (c). Increase in absorbance followed at 437 nm due to the action of tyrosinase (0.1 mg/mL) on thymol 1.5 mM and 0.083 mM (3I6MC) in 30 mM phosphate buffer (pH 7.0). Curve (d). Increase in absorbance at 437 nm due to the action of tyrosinase (0.1 mg/mL) on these monophenols: guaiacol 1.5 mM, carvacrol 1.5 mM, and thymol 1.5 mM in the absence of MBTH (guaiacol) or 3I6MC (carvacrol and thymol). (**B**) Spectrophotometric recording on the action of tyrosinase on eugenol or isoeugenol in the presence of MBTH. Curve (a). Eugenol 1 mM in the presence of MBTH 1 mM N,N′ dimethylformamide 2% in 50 mM phosphate buffer (pH 7.0) at 540 nm. Curve (b). Isoeugenol 1 mM in the presence of MBTH 1 mM N,N′ dimethylformamide 2% in 50 mM phosphate buffer (pH 7.0) at 540 nm. Curve (c). Increase in absorbance followed at 540 nm in the action of tyrosinase (0.1 mg/mL) on eugenol 1 mM or isoeugenol 1 mM in the absence of MBTH. Inset. Curve (d). Increase in absorbance at 480 nm in the action of tyrosinase on ferulic acid 1.5 mM in the presence of 2-methoxycatechol 0.09 mM in 30 mM phosphate buffer (pH 7.0). Curve (e). Increase in absorption followed at 480 nm due to the action of tyrosinase (0.1 mg/mL) on ferulic acid 1.5 mM in the absence of 3-methoxycatechol.

In the case of ortho-substituted phenols such as thymol, carvacrol, guaiacol, eugenol, isoeugenol, and ferulic acid (Figure 1), it has been shown that in the presence of hydrogen peroxide or catalytic *o*-diphenol, these compounds behave as alternative substrates of the enzyme [12,13]. Gowda and Paul showed with tyrosinase extracted from *Dolichos lablab* that in the presence of catechol, the enzyme was active on ferulic acid (FA) (Figure 1). Figure 2 of reference [13] shows the accumulation of the FA oxidation product with time. It is noted that the accumulation of *o-*quinone occurs with a delayed period that increases with an increasing substrate concentration and decreases with an increasing enzyme concentration; this corresponds to the test of the tyrosinase monophenolase activity [3].

By applying molecular docking, it seems evident that in the case of mushroom tyrosinase, these molecules are located in the active center of the enzyme in a position such that the distance of the oxygen of the peroxide group in oxy-tyrosinase to the ortho position of the phenolic hydroxyl is suitable for the electrophilic attack to occur and therefore carry out the hydroxylation reaction. Thus, ortho-substituted monophenols can bind to met-tyrosinase in an inhibiting way and to oxy-tyrosinase catalytically as alternative substrates.

## 2. Results and Discussion

In this section, we will consider the studies carried out with ortho-substituted phenols under the approach of tyrosinase inhibitors or substrates, and simulation studies by the docking of these ligands to the enzyme will be used as an aid.

### 2.1. The Apparent Inhibition in the Action of Tyrosinase on L-Dopa in the Presence of Ortho-Substituted Phenols

Ortho-substituted phenols such as ferulic acid (FA) have high potential as antioxidants [14,15]. Regarding FA, its protection against hydroxyl and peroxyl radicals has been studied in neural cell culture [16]. It has been described that FA is a tyrosinase inhibitor by studying the inhibition of L-dopa oxidation; the inhibition has been described as competitive [17]. Isoferulic acid (IFA) was also described as an inhibitor of mushroom tyrosinase with ${IC}_{50}\;$ values of 0.13 mM and 0.35 mM for the monophenolase and diphenolase activities of the enzyme [18]. Other ortho-substituted phenols (thymol, carvacrol) have been described in the literature as inhibitors of the melanin biosynthesis pathway, but in this case, it was proposed that they do not act directly on the enzyme but do so at the level of the chemical reactions that occur from *o*-dopaquinone to dopachrome [19].

In a recent work, FA has been described as an inhibitor of tyrosinase; according to this publication, FA apparently behaves as a competitive inhibitor of the enzyme [20]. The authors, based on their data shown in Figure 1a,b of [20], propose that it is a competitive inhibition. 

**Scheme 3:**
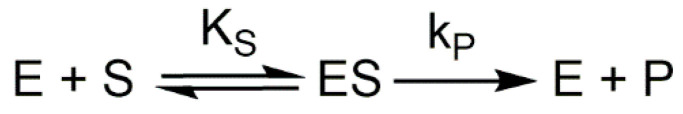
Michaelis–Menten mechanism. Where S is the substrate, P is the product, and E is the enzyme. $K_{S}$ is the dissociation constant of the ES complex, and $\;k_{P}$ is the catalytic constant.

The action of a Michaelian enzyme on its substrate is expressed as follows (Scheme 3):

The equation of the rate of accumulation of the product P is as follows:

(1) $$V_{0}^{P}=\frac{V_{max}^{P}{\left[S\right]}_{0}}{K_{S}^{S}+{\left[S\right]}_{0}}$$

**Scheme 4:**
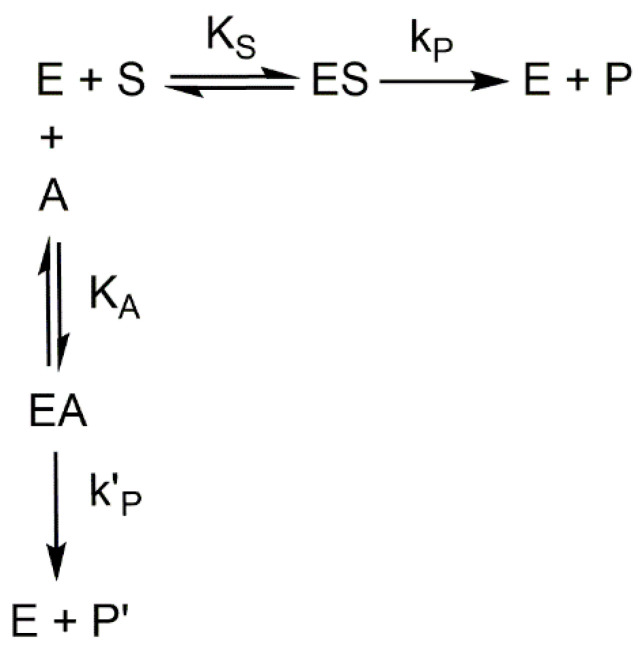
Michaelis–Menten mechanism in the presence of an alternative substrate, A. P′ is the product of A.

If the enzyme acts on two substrates S and A, the scheme would be as follows:

In Scheme 4, if S is L-dopa and P is dopachrome, in the presence of substrate A that is studied as an inhibitor, such as FA, if ${\left[S\right]}_{0}>{\left[A\right]}_{0}yk_{P}>k_{P}^{\prime }$, the concentration of P is greater than that of P′. Regarding kinetics, although distorted, apparently the Lineveaver–Burck graphs may correspond to competitive inhibition, according to Equation (2). However, if ${IC}_{50}\;$ and $K_{I}$ are determined, according to Equation (3), ${IC}_{50}$ should be greater than $K_{I}$, but the data obtained indicate the opposite. Thus, the proposed values for the ${IC}_{50}$ of the FA and the $K_{I}$ value turn out to be 0.23 µM and 6.8 µM, respectively [20].

(2) $$V_{0}^{P}=\frac{V_{max}^{P}{\left[S\right]}_{0}}{K_{S}^{S}\left(1+\frac{{\left[A\right]}_{0}}{K_{A}}\right)+{\left[S\right]}_{0}}$$

Equation (2) corresponds to a competitive inhibition apparently, which in turn corresponds to Figure 1 [20].

The kinetic data described in [20] do not meet Equation (3) since ${IC}_{50}$ < $K_{I}$

(3) $${IC}_{50}=(1+n)K_{I}^{app}$$

where IC_50_ is the inhibitor concentration required to achieve 50% inhibition at a substrate concentration ${\left[S\right]}_{0}$, and “n” is the ratio between the substrate concentration and the $K_{S}^{S}$.

(4) $$n=\frac{{\left[S\right]}_{0}}{K_{S}^{S}}$$

However, although the Lineweaver–Burk graphs apparently correspond to competitive inhibition, the ${IC}_{50}$ data with respect to the inhibition constant $K_{I}$ do not comply with the kinetic tests since ${IC}_{50}$ values of 0.23 µM are described for the FA, with a $K_{I}$ value of 6.8 µM [20]. The kinetic analysis predicts that the values of ${IC}_{50}$ should be greater than those of $K_{I}$ according to Equation (3). This is not true in the data described above, and therefore these compounds do not behave as pure competitive inhibitors; this inhibition is only apparent [21]. More recently, a review has been published on the action of FA and its derivatives as potent tyrosinase inhibitors, indicating that the inhibition capacity is due to the presence of double bond conjugation as a side chain in is molecule [22]. We, based on published data and those described in Figure 2, propose that FA is an alternative substrate of the enzyme [23].

### 2.2. Characterization of Ortho-Substituted Phenols as Tyrosinase Substrates

In the literature, ortho-substituted phenols have often been described as tyrosinase inhibitors. The monophenolase activity of tyrosinase from the kinetic point of view is complex. In the case of the activity on L-tyrosine, the enzymatic activity followed experimentally by the accumulation of dopachrome is preceded by a delay period, which is the time that the enzyme takes in its action on l-tyrosine to accumulate in the medium a certain amount of *o-*diphenol (L-dopa) [3,24,25]; this, from a chemical point of view, is possible since *o*-dopaquinone evolves chemically, accumulating *o-*diphenol in the medium. In the case of *o*-substituted phenols, the *o*-quinone originated by the oxy-tyrosinase form does not evolve, accumulating *o*-diphenol in the medium, and therefore the enzyme does not show activity. The only possibility that tyrosinase has of showing catalytic activity on is by the following:

1. Achieve the transition from E_m_ to E_oxy_ by adding hydrogen peroxide according to E_m_ + H_2_O_2_ ⇄ Eoxy (Scheme 1). Hydrogen peroxide causes the transformation of the met-tyrosinase form (inactive on monophenols) to the oxy-tyrosinase form (active on monophenols).
2. Study the action of tyrosinase on monophenols such as thymol and carvacrol in the presence of their corresponding *o*-diphenol: 3-isopropyl-6-methylcatechol or 3-methoxycatechol in the case of guaiacol. In these cases, it is the added *o*-diphenol that causes the transition from met-tyrosinase to oxy-tyrosinase, according to E_m_ + D⇄ E_m_D → Q + E_d_ + O_2_ ⇄ Eox. The kinetic parameters described in Table 1 are obtained [26].
3. Study the action of tyrosinase on *o*-substituted monophenol in the presence of an *o*-diphenol such as L-dopa.

**Scheme 5:**
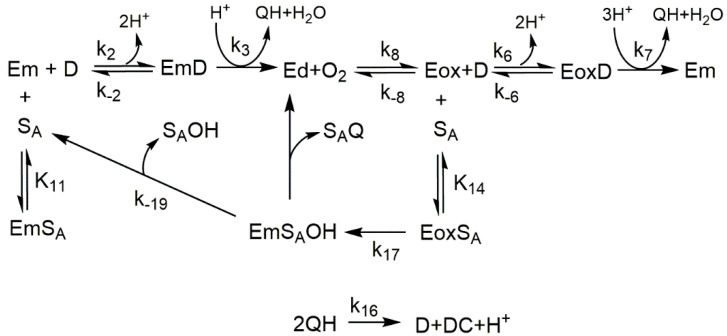
Mechanism of action of tyrosinase on L-dopa in the presence of an alternative substrate (monophenol). Where S_A_ is the alternative substrate, S_A_OH is the hydroxylated alternative substrate, S_A_Q is the *o*-quinone of the hydroxylated alternative substrate, E_m_S_A_ is the complex of E_m_ with S_A_, and E_m_S_A_OH is the complex of Em and hydroxylated S_A_.

Scheme 5 shows the action of tyrosinase on *o*-substituted monophenols in the presence of L-dopa. In this case, it is L-dopa that carries out the step from meta-tyrosinase to oxy-tyrosinase.

4. To study the action of tyrosinase on *o*-subtitled monophenols in the presence of catechol, the mechanism is similar to cases 1, 2, and 4. The FA was studied under this experimental approach by measuring the increase in absorbance at λ = 480 nm; thus, the kinetic parameters shown in Table 1 were determined [13].
5. Study the action of the enzyme on ortho-substituted phenol in the presence of hydrogen peroxide (Scheme 1), or a nucleophile like MBTH and the nucleophile MBTH in order to increase the spectrophotometric signal, since the adducts that originate from the attack of MBTH on *o*-quinone have a very high molar absorptivity coefficient (Scheme 2) [27]. Under this approach, FA was studied [27], thus characterizing the value $K_{M}^{FA}$ (see Table 1).

Figure 2 shows the action of tyrosinase on these compounds in the presence of *o*-diphenol in catalytic concentrations or in the presence of MBTH. It is revealed that all of them behave as tyrosinase substrates.

Table 1 shows the data obtained from the experiments carried out with some ortho-substituted phenols. In the case of FA, catechol is used as *o*-diphenol. Due to the strong steric hindrance of these molecules, the $K_{M}$ values are high, so it has not been possible to separate the kinetic parameters $K_{M}$ and $k_{cat}$. This could only be carried out in the case of guaiacol (K_M_ = 4.65 ± 0.39 mM and k_cat_ = 5.4 ± 0.14 s^−1^) [26] and in the case of thymol and carvacrol; the ratio $k_{cat}/K_{M}$, that is, the second order constant, resulted in being 161 ± 4 M^−1^ s^−1^ and 95 ± 7 M^−1^ s^−1^, respectively. In the case of eugenol and isoeugenol, MBTH was added to the reaction medium; this allowed *o*-diphenol to accumulate in the medium through the nucleophilic attack on the *o*-quinone generated by the enzyme (see Supplementary Material in [26]).

### 2.3. Docking Studies of the Compounds Described in This Work to Tyrosinase

Ortho-substituted phenols can have a large steric hindrance in their binding to the active site of tyrosinase; furthermore, the hydroxylation reaction requires an adequate distance between the oxygen of the peroxide bridge of oxy-tyrosinase and the free ortho position to the hydroxyl group on the ring benzene. The values obtained for the studied compounds are shown in Table 2.

**Figure 3:**
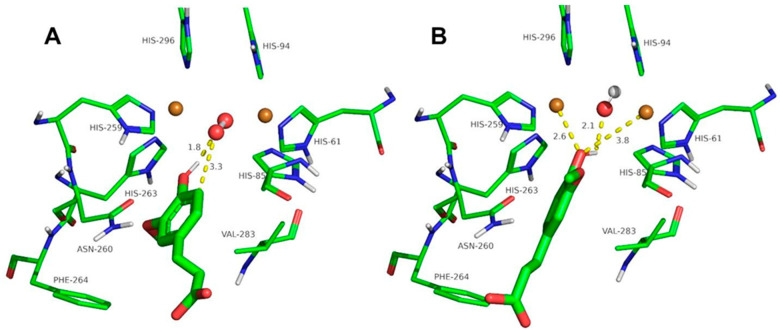
Docking of ferulic acid to *oxy*-tyrosinase (**A**) and to *met*-tyrosinase (**B**). The atom colors are as follows: carbon = green, oxygen = red, nitrogen = blue, copper = brown, and hydrogen = white. Ferulic acid is shown in thick sticks, and distances are shown in yellow dashed lines.

**Figure 4:**
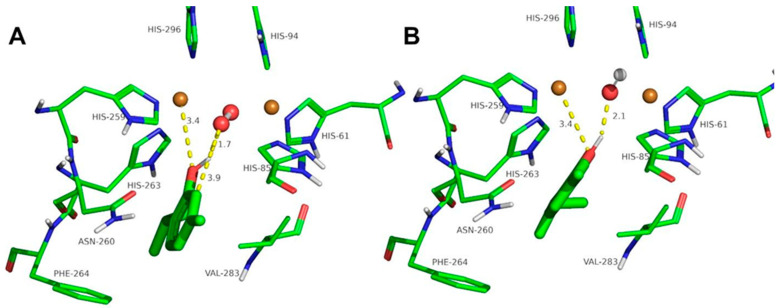
Docking of thymol to *oxy*-tyrosinase (**A**) and to *met*-tyrosinase (**B**). The atom colors are as follows: carbon = green, oxygen = red, nitrogen = blue, copper = brown, and hydrogen = white. Thymol is shown in thick sticks, and distances are shown in yellow dashed lines.

**Figure 5:**
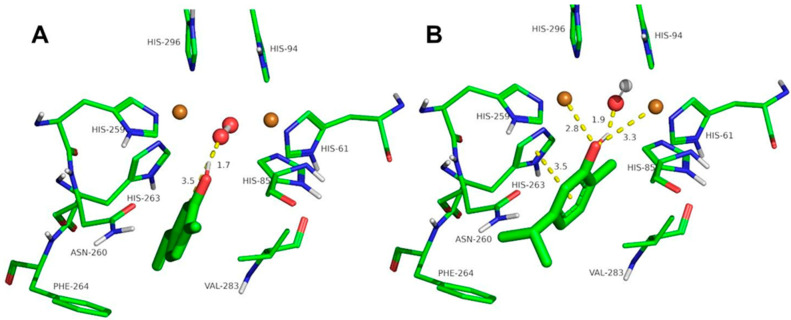
Docking of carvacrol to *oxy*-tyrosinase (**A**) and to *met*-tyrosinase (**B**). The atom colors are as follows: carbon = green, oxygen = red, nitrogen = blue, copper = brown, and hydrogen = white. Carvacrol is shown in thick sticks, and distances are shown in yellow dashed lines.

**Figure 6:**
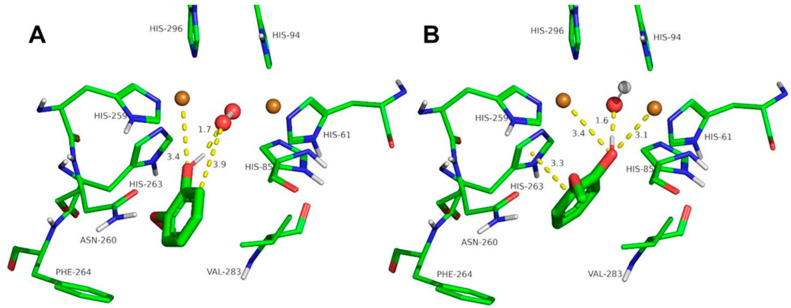
Docking of guaiacol to *oxy*-tyrosinase (**A**) and to *met*-tyrosinase (**B**). The atom colors are as follows: carbon = green, oxygen = red, nitrogen = blue, copper = brown, and hydrogen = white. Guaiacol is shown in thick sticks, and distances are shown in yellow dashed lines.

**Figure 7:**
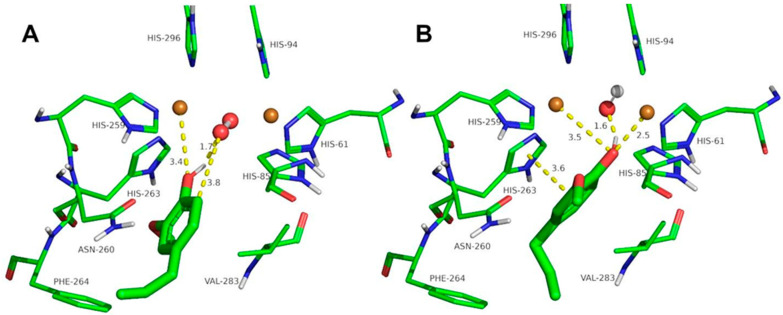
Docking of eugenol to *oxy*-tyrosinase (**A**) and to *met*-tyrosinase (**B**). The atom colors are as follows: carbon = green, oxygen = red, nitrogen = blue, copper = brown, and hydrogen = white. Eugenol is shown in thick sticks, and distances are shown in yellow dashed lines.

**Figure 8:**
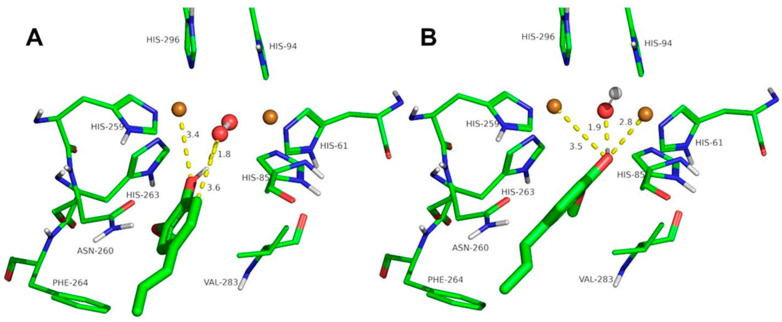
Docking of isoeugenol to *oxy*-tyrosinase (**A**) and to *met*-tyrosinase (**B**). The atom colors are as follows: carbon = green, oxygen = red, nitrogen = blue, copper = brown, and hydrogen = white. Isoeugenol is shown in thick sticks, and distances are shown in yellow dashed lines.

The docking of the ortho-substituted phenols at the binuclear copper active site of tyrosinase is shown in Figure 3, Figure 4, Figure 5, Figure 6, Figure 7 and Figure 8. The docking of FA at the binuclear copper active site of tyrosinase is shown in Figure 3. In the oxy form (Figure 3A), the hydrogen atom of the hydroxyl group of FA acid is at 1.8 Å from the oxygen atom of the peroxide ion, and the oxygen atom of the ligand is at 3.3 Å from a copper ion. The peroxide ion is at 3.3 Å from the ortho carbon to the phenolic group of the ligand, and the hydroxylation of FA could be plausible with a K_d_ of 7.8 mM (Table 2). In the met form (Figure 3B), the hydrogen atom of the hydroxyl group of FA is at 2.1 Å, and the oxygen atom of the phenolic group is at 2.6 and 3.8 Å from the copper ions. This docking conformation could also be an inhibitory interaction of the ligand with a K_d_ of 4.8 mM (Table 2). 

The docking of other ortho-substituted phenols molecules, such as thymol, carvacrol, guaiacol, eugenol, and isoeugenol, has been carried out, and their corresponding docking poses are shown in Figure 4, Figure 5, Figure 6, Figure 7 and Figure 8. All the docking results are summarized in Table 2. It can be seen that all the monophenols studied behave in a similar way; they can reach the ligand binding pocket in both the oxy and met forms. However, only in the oxy form could they be catalyzed (hydroxylated), while in the met form they would act as inhibitors.

Ortho-substituted phenols may exhibit a high steric hindrance to binding to the active site of tyrosinase. On the other hand, the hydroxylation reaction in the ortho position to the hydroxyl group of the benzene ring requires an appropriate distance from the peroxide group of oxy-tyrosinase to the carbon atom in the ortho position to the hydroxyl group. The docking results of the different compounds studied in this work are shown in Table 2. It is important to note that these compounds are located in the active site of tyrosinase in positions that allow for the electrophilic attack of oxygen peroxide on the carbon atom and the hydroxyl phenolic ortho.

Despite the steric hindrance that these molecules may present, Figure 3, Figure 4, Figure 5, Figure 6, Figure 7 and Figure 8 and Table 2 show how monophenols can bind to the met and oxy forms of the enzyme. The met form is inactive; on the contrary, the oxy form is active since the distance from the peroxide group to the ortho position of the phenolic group is adequate for the electrophilic attack to be carried out on the substrate molecule.

## 3. Material and Methods

### 3.1. Enzyme Source

Mushroom tyrosine or polyphenol oxidase (*o*-diphenol, oxygen-oxidoreductase, EC 1.14.18.1, 4276 U/mg) was supplied by Sigma (Madrid, Spain). The enzyme was purified as previously described [28]. The protein concentration was determined by Bradford′s method using bovine serum albumin as standard [29].

### 3.2. Reagents

4-Hydroxyphenylalanine (L-tyrosine), 3,4-dihydroxy-phenylalanine (L-dopa), 2-isopropyl-5-methylphenol (thymol), 5-isoropyl-2-methilphenol (carvacrol), 2-methoxyphenol (guaiacol), 4-allyl-2-methoxyphenol (eugenol), 2-methoxy-4-propenylphenol (isoeugenol), 3-methoxycatechol (3MC), 3-isopropyl-6-methylcatecol (316MC), 3-methyl-2-benzothiazolinone hydrazone hydrochloride hydrate (MBTH), N,N-dimethylformamide (DMF), ammonium acetate, and 4-tert-butylcatechol were all from Sigma (Madrid, Spain). The corresponding structures of the monophenols studied are shown in (Figure 1). The chemicals were of analytical grade.

### 3.3. Spectrophotometric Assays

Spectrophotometric assays of the enzymatic activity of tyrosinase in its action on different monophenols were followed at maximum absorbance in the visible spectrophotometric region of the *o*-quinone corresponding to the monophenol under study [30]. Figure 2 shows the measurement conditions for each substrate.

### 3.4. Computational Simulation of Interaction between Ortho-Substituted Phenols to Met-Tyrosinase and Oxy-Tyrosinase

The chemical structures of all ligands used were obtained from the PubChem Substance and Compound database [31] through the following chemical structure identifiers: 6989 (thymol), 10364 (carvacrol), 460 (guaiacol), 3314 (eugenol), 853433 (isoeugenol), 445858 (ferulic acid), and 1794427 (chlorogenic acid).

The molecular structure, the deoxy-form of tyrosinase from *Agaricus bisporus*, was taken from the Protein Databank (PDB ID:2Y9W, Chain A) [32]. The input protein structure for docking was prepared by adding all hydrogen atoms and removing water molecules. The *met* and *oxy* forms of tyrosinase were built, as previously described [33], by slightly modifying the binuclear copper-binding site to comply with the *met* and *oxy* forms crystallized from *Streptomyces castaneoglobispours* corresponding to Protein Data Bank entries 2AHK and 1WX2, respectively. 

Gasteiger’s partial charges and rotatable bonds were assigned by AutoDockTools4 software [34,35]. AutoDock 4.2.6 software [35] was used for protein-ligand docking calculations. The scoring function in AutoDock is based on the United Atom version of the AMBER force field. Semi-flexible docking of the ligands allows for the sampling of various docking conformations while the receptor protein remains rigid. 

The receptor docking site was in the active site of tyrosinase, where the molecular docking of all ligands was studied. Grid parameter files were built using AutoGrid 4.2.6 [36]. The Lamarkian Genetic Algorithm was chosen to explore the space of active binding to search for the best conformers. Genetic algorithm was run for 2000 sets, and the maximum number of energy evaluations was set to 25,000,000 in each run. The maximum number of generations simulated during each GA run was set to 270,000. The rates of the gene mutation and crossover were set to 0.02 and 0.8, respectively. Other docking parameters were used as in [37]. PyMOL 2.3.0 [38] was used for 3D visualization of molecular structures, to analyze the docking results, and to prepare the figures of the docked conformations.

## 4. Conclusions

In conclusion, tyrosinase is an enzyme with broad substrate specificity, both for monophenols and *o*-diphenols [39]. The docking data shown for these ortho-substituted phenols indicate that these compounds can be substrates for tyrosinase, as described experimentally. In the case of ortho-substituted monophenols (ferulic acid, thymol, carbacrol, guaiacol, eugenol, and isoeugenol), their binding to the met-tyrosinase form is inactive; however, there is activity in the oxy-tyrosinase form. And whenever we consider molecules with a free phenolic hydroxyl group, it must be shown that they are not alternative substrates for tyrosine and thus avoid possible interferences in the composition of cosmetics and depigmenting agents.

## Figures and Tables

**Table 1:** Kinetic parameters and constants characterizing the oxidation of thymol, carvacrol, guaiacol, and ferulic acid by tyrosinase.

Substrate	Enzyme	k_cat_ (S^−1^)	K_M_ (mM)	k_cat_/K_M_ (M^−1^ S^−1^)	References
Thymol	Mushroom (*Agaricus Bisporus*)			161 ± 4	[26]
Carvacrol	Mushroom (*Agaricus Bisporus*)			95 ± 7	[26]
Guaiacol	Mushroom (*Agaricus Bisporus*)	5.40 ± 0.14	4.65 ± 0.39	1160 ± 101	[26]
Ferulic acid	Field Bean (*Dolichos lablab*)		0.09		[13]
Ferulic acid	Grean Bean (*Phaseolus vulgaris*) extract		0.23		[27]

**Table 2:** Molecular docking dissociation constants (mM) and distances (Å) for oxy and met forms of tyrosinase. Distances greater than 4 Å have not been considered.

Ligand	Oxy Form					Met Form			
	K_d_	d(O_2_-HO)	d(O_2_-oC)	d(Cu-OH)	d(π-π)	K_d_	d(OH-OH)	d(Cu-OH)	d(π-π)
Ferulic acid	7.8	1.8	3.3	3.3	-	4.8	2.1	2.6	-
Thymol	0.61	1.7	3.9	3.4	-	0.92	2.1	3.4	-
Carvacrol	0.9	1.7	3.5	-	-	0.84	1.9	2.8	3.5
Guaiacol	7.3	1.7	3.9	3.4	-	2.2	1.6	3.1	3.3
Eugenol	3.9	1,7	3.8	3.4	-	0.63	1.6	2.5	3.6
Isoeugenol	3	1.8	3,6	3.4	-	2.4	1.9	2.8	-

d(O_2_-HO): minimum distance from O atom of peroxide group of tyrosinase to H atom of phenolic group of ligand. d(O_2_-oC): minimum distance from O atom of peroxide group of tyrosinase to carbon atom of ligand in ortho position with respect to the phenolic group. d(Cu-OH): minimum distance from a Cu atom of tyrosinase to O atom of phenolic group of ligand. d(π-π): distance from the center of the imidazole ring of H263 to the center of the aromatic ring of the ligand. d(OH-OH): minimum distance from H atom of hydroxyl group of tyrosinase to O atom of phenolic group of ligand.

## Data Availability

Data is contained within the article.

## References

[1] Sánchez-Ferrer Á., Rodríguez-López J.N., García-Cánovas F., García-Carmona F. (1995). Tyrosinase: A comprehensive review of its mechanism. Biochim. Biophys. Acta—Protein Struct. Mol. Enzymol..

[2] Solomon E.I., Sundaram U.M., Machonkin T.E. (1996). Multicopper oxidases and oxygenases. Chem. Rev..

[3] Molina F.G., Muñoz J.L., Varón R., López J.N.R., Cánovas F.G., Tudela J. (2007). An approximate analytical solution to the lag period of monophenolase activity of tyrosinase. Int. J. Biochem. Cell Biol..

[4] García Molina M.d.M., Muñoz Muñoz J.L., Martinez Ortiz F., Martinez J.R., García Ruiz P.A., Rodriguez López J.N., García Cánovas F. (2014). Tyrosinase-catalyzed hydroxylation of hydroquinone, a depigmenting agent, to hydroxyhydroquinone: A kinetic study. Bioorg. Med. Chem..

[5] Ortiz-Ruiz C.V., Ballesta de los Santos M., Berna J., Fenoll J., Garcia-Ruiz P.A., Tudela J., Garcia-Canovas F. (2015). Kinetic characterization of oxyresveratrol as a tyrosinase substrate. IUBMB Life.

[6] Ortiz-Ruiz C.V., Berna J., Rodriguez-Lopez J.N., Tomas V., Garcia-Canovas F. (2015). Tyrosinase-Catalyzed Hydroxylation of 4-Hexylresorcinol, an Antibrowning and Depigmenting Agent: A Kinetic Study. J. Agric. Food Chem..

[7] Garcia-Jimenez A., García-Molina F., Teruel-Puche J.A., Saura-Sanmartin A., Garcia-Ruiz P.A., Ortiz-Lopez A., Rodríguez-López J.N., Garcia-Canovas F., Munoz-Munoz J. (2018). Catalysis and inhibition of tyrosinase in the presence of cinnamic acid and some of its derivatives. Int. J. Biol. Macromol..

[8] Garcia-Jimenez A., Teruel-Puche J.A., Ortiz-Ruiz C.V., Berna J., Tudela J., Garcia-Canovas F. (2016). 4-n-butylresorcinol, a depigmenting agent used in cosmetics, reacts with tyrosinase. IUBMB Life.

[9] Garcia-Jimenez A., Teruel-Puche J.A., Berna J., Rodriguez-Lopez J.N., Tudela J., Garcia-Ruiz P.A., Garcia-Canovas F. (2016). Characterization of the action of tyrosinase on resorcinols. Bioorg. Med. Chem..

[10] García Molina M.d.M., Berna J., Muñoz-Muñoz J.L., García-Ruiz P.A., Moreno M.G., Martinez J.R., Garcia-Canovas F. (2014). Action of tyrosinase on hydroquinone in the presence of catalytic amounts of o-diphenol. A kinetic study. React. Kinet. Mech. Catal..

[11] Muñoz-Muñoz J.L., García-Molina F., Varón R., Tudela J., García-Cánovas F., Rodríguez-López J.N. (2009). Generation of hydrogen peroxide in the melanin biosynthesis pathway. Biochim. Biophys. Acta—Proteins Proteom..

[12] Muñoz-Muñoz J.L., Berna J., Rodríguez-López J.N., Varón R., García-Cánovas F., Mary of the Sea GARCÍA-MOLINA (2013). Hydrogen Peroxide Helps in the Identification of Monophenols as Possible Substrates of Tyrosinase. Biosci. Biotechnol. Biochem..

[13] Gowda L.R., Paul B. (2002). Diphenol Activation of the Monophenolase and Diphenolase Activities of Field Bean (Dolichos lablab) Polyphenol Oxidase. J. Agric. Food Chem..

[14] Graf E. (1992). Antioxidant potential of ferulic acid. Free Radic. Biol. Med..

[15] Rosazza J.P.N., Huang Z., Dostal L., Volm T., Rousseau B. (1995). Review: Biocatalytic transformations of ferulic acid: An abundant aromatic natural product. J. Ind. Microbiol. Biotechnol..

[16] Kanski J., Aksenova M., Stoyanova A., Butterfield D.A. (2002). Ferulic acid antioxidant protection against hydroxyl and peroxyl radical oxidation in synaptosomal and neuronal cell culture systems in vitro: Structure-activity studies. J. Nutr. Biochem..

[17] Gong S.Z., Cheng J.A., Yang Z.R. (2005). Inhibitory effect of ferulic acid on oxidation of L-DOPA catalyzed by mushroom tyrosinase. Chin. J. Chem. Eng..

[18] Gong S., Yin M., Yun Z. (2013). Kinetics of inhibitory effect of isoferulic acid on mushroom tyrosinase. J. Cosmet. Sci..

[19] Satooka H., Kubo I. (2011). Effects of Thymol on Mushroom Tyrosinase-Catalyzed Melanin Formation. J. Agric. Food Chem..

[20] Shojazadeh T., Zolghadr L., Gharaghani S., JafarKhani S., Molaabasi F., Piri H., Gheibi N. (2023). New insights into the inhibitory effect of phenol carboxylic acid antioxidants on mushroom tyrosinase by molecular dynamic studies and experimental assessment. J. Biomol. Struct. Dyn..

[21] García-Molina P., Garcia-Molina F., Teruel-Puche J.A., Rodriguez-Lopez J.N., Garcia-Canovas F., Muñoz-Muñoz J.L. (2022). The relationship between the IC50 values and the apparent inhibition constant in the study of inhibitors of tyrosinase diphenolase activity helps confirm the mechanism of inhibition. Molecules.

[22] Alifah L.H.N., Jatmika C., Hayun H. (2024). Exploration of Ferulic Acid and Its Derivatives as Potent Anti-Tyrosinase: A Systematic Review. Egypt. J. Chem..

[23] Ortiz-Ruiz C.V., Garcia-Molina M.d.M., Serrano J.T., Tomas-Martinez V., Garcia-Canovas F. (2015). Discrimination between Alternative Substrates and Inhibitors of Tyrosinase. J. Agric. Food Chem..

[24] Ros J., Rodríguez-López J., García-Cánovas F. (1994). Tyrosinase: Kinetic analysis of the transient phase and the steady state. Biochim. Biophys. Acta—Protein Struct. Mol. Enzymol..

[25] Fenoll L.G., Rodríguez-López J.N., García-Sevilla F., García-Ruiz P.A., Varón R., García-Cánovas F., Tudela J. (2001). Analysis and interpretation of the action mechanism of mushroom tyrosinase on monophenols and diphenols generating highly unstable o-quinones. Biochim. Biophys. Acta—Protein Struct. Mol. Enzymol..

[26] Garcia-Molina M.d.M., Muñoz-Muñoz J.L., Garcia-Molina F., García-Ruiz P.A., Garcia-Canovas F. (2012). Action of Tyrosinase on Ortho-Substituted Phenols: Possible Influence on Browning and Melanogenesis. J. Agric. Food Chem..

[27] Morosanova M., Fedorov A., Morosanova E. (2018). Crude Plant Extracts Mediated Polyphenol Oxidation Reactions in Presence of 3-Methyl-2-Benzothiazolinone Hydrazone for Determination of Total Polyphenol Content in Beverages. Curr. Anal. Chem..

[28] Rodríguez-López J.N., Fenoll L., García-Ruiz P.A., Varón R., Tudela J., Thorneley R.N.F., García-Cánovas F. (2000). Stopped-flow and steady-state study of the diphenolase activity of mushroom tyrosinase. Biochemistry.

[29] Bradford M.M. (1976). A rapid and sensitive method for the quantitation of microgram quantities of protein utilizing the principle of protein-dye binding. Anal. Biochem..

[30] García-Molina F., Muñoz-Muñoz J.L., Varón R., Rodríguez-López J.N., García-Cánovas F., Tudela J. (2007). A review on spectrophotometric methods for measuring the monophenolase and diphenolase activities of tyrosinase. J. Agric. Food Chem..

[31] Kim S., Thiessen P.A., Bolton E.E., Chen J., Fu G., Gindulyte A., Han L., He J., He S., Shoemaker B.A. (2016). PubChem substance and compound databases. Nucleic Acids Res..

[32] Ismaya W.T., Rozeboom H.J., Weijn A., Mes J.J., Fusetti F., Wichers H.J., Dijkstra B.W. (2011). Crystal structure of *Agaricus bisporus* mushroom tyrosinase: Identity of the tetramer subunits and interaction with tropolone. Biochemistry.

[33] Maria-Solano M.A., Ortiz-Ruiz C.V., Muñoz-Muñoz J.L., Teruel-Puche J.A., Berna J., Garcia-Ruiz P.A., Garcia-Canovas F. (2016). Further insight into the pH effect on the catalysis of mushroom tyrosinase. J. Mol. Catal. B Enzym..

[34] Sanner M.F. (1999). Python: A programming language for software integration and development. J. Mol. Graph. Model..

[35] Morris G.M., Huey R., Lindstrom W., Sanner M.F., Belew R.K., Goodsell D.S., Olson A.J. (2009). AutoDock4 and autoDockTools4: Automated docking with selective receptor flexibility. J. Comput. Chem..

[36] Huey R., Morris G.M., Olson A.J., Goodsell D.S. (2007). A semiempirical free energy force field with charge-based desolvation. J. Comput. Chem..

[37] García-Molina P., García-Molina F., Teruel-Puche J.A., Rodríguez-López J.N., García-Cánovas F., Muñoz-Muñoz J.L. (2022). Considerations about the kinetic mechanism of tyrosinase in its action on monophenols: A review. Mol. Catal..

[38] Schrödinger L. (2015). The PyMOL Molecular Graphics System, Version 2.3.

[39] Espín J.C., Varón R., Fenoll L.G., Gilabert M.A., García-Ruíz P.A., Tudela J., García-Cánovas F. (2000). Kinetic characterization of the substrate specificity and mechanism of mushroom tyrosinase. Eur. J. Biochem..

